# Difference in Prognosis between Continuation and Discontinuation of A 5-Month Cardiac Rehabilitation Program in Outpatients with Heart Failure with Preserved Ejection Fraction

**DOI:** 10.3390/jcm10153306

**Published:** 2021-07-27

**Authors:** Hidetaka Morita, Yasunori Suematsu, Kai Morita, Yuiko Yano, Maaya Sakamoto, Takuro Matsuda, Kouji Kaino, Reiko Teshima, Nobuyuki Ura, Masaomi Fujita, Rie Tazawa, Hironari Nakagawa, Ken Kitajima, Satoshi Kamada, Kanta Fujimi, Shin-ichiro Miura

**Affiliations:** 1 Department of Cardiology, Fukuoka University Hospital, 7-45-1 Nanakuma, Jonan-Ku, Fukuoka 814-0180, Japan; h.morita.us@adm.fukuoka-u.ac.jp (H.M.); ysuematsu@fukuoka-u.ac.jp (Y.S.); kurisuchan0924@gmail.com (K.M.); yuicom0109@hotmail.co.jp (Y.Y.); yaamami@gmail.com (M.S.); 2 Center for Cardiac Rehabilitation, Fukuoka University Hospital, 7-45-1 Nanakuma, Jonan-Ku, Fukuoka 814-0180, Japan; gd050010@yahoo.co.jp (T.M.); kenkitaj@fukuoka-u.ac.jp (K.K.); 3 Department of Rehabilitation, Fukuoka University Hospital, 7-45-1 Nanakuma, Jonan-Ku, Fukuoka 814-0180, Japan; goeminence@yahoo.co.jp (K.K.); reiko.dai.222@icloud.com (R.T.); cdhcn430@yahoo.co.jp (N.U.); omiomi1973@gmail.com (M.F.); k1213026@gmail.com (H.N.); kmd@fukuoka-u.ac.jp (S.K.); 4 Division of Nutrition, Fukuoka University Hospital, 7-45-1 Nanakuma, Jonan-Ku, Fukuoka 814-0180, Japan; tazawa0220@fukuoka-u.ac.jp; 5 Postgraduate Clinical Training Center, Fukuoka University Hospital, 7-45-1 Nanakuma, Jonan-Ku, Fukuoka 814-0180, Japan; 6 Department of Cardiology, Fukuoka University Nishijin Hospital, 15-4 Sohara Sawara-Ku, Fukuoka 814-8522, Japan

**Keywords:** preserved ejection fraction, cardiac rehabilitation program, continued group, discontinued group

## Abstract

Background: Cardiac rehabilitation (CR) is a requisite component of care for patients with heart failure (HF). We aimed to evaluate the clinical outcomes in outpatients with HF with preserved ejection fraction (HFpEF) compared to those in patients with non-HFpEF who did and did not continue a 5-month CR program. Methods: 173 outpatients with HF who participated in a 5-month CR program were registered. We divided them into two groups: HFpEF (*n* = 84, EF 63 ± 7%) and non-HFpEF (*n* = 89, EF 31 ± 11%). We further divided the patients into those who continued the CR program (continued group) and those who did not (discontinued group) in the HFpEF and non-HFpEF groups. The clinical outcomes at 5 months were compared among the groups. Results: There were no significant differences in patient characteristics at baseline between the continued and discontinued groups in the HFpEF and non-HFpEF groups except for % diabetes mellitus in the non-HFpEF group. The rates of all-cause death and hospital admissions in the continued group in both the HFpEF and non-HFpEF groups were significantly lower than those in the discontinued group. The all-cause death and hospital admissions in each group were independently associated with the continuation of the CR program. Conclusions: The continuation of a 5-month CR program was associated with the prevention of all-cause death and hospital admissions in both the HFpEF and non-HFpEF groups.

## 1. Introduction

Cardiac rehabilitation (CR) is a requisite component of care for patients with heart failure (HF) and a class A recommendation in several guidelines [1,2]. CR improves cardiorespiratory function and quality of life (QOL) and reduces mortality in patients with HF with reduced left ventricular ejection fraction (HFrEF) [3,4]. Although we treat many patients with HF with preserved ejection fraction (HFpEF) [5], patients with HFpEF and HFrEF have almost the same survival rates [6]. Furthermore, no pharmacotherapy has been shown to improve the survival rate in patients with HFpEF [7,8,9,10,11].

We have studied CR for more than eight years and reported some beneficial effects in patients with cardiovascular diseases (CVD) [12,13,14,15,16,17]. We previously reported that a CR program significantly reduced blood pressure (BP) and visit-to-visit variability in BP, and patients with mild to moderate chronic kidney disease (CKD) who participated in our CR program showed an increase in their estimated glomerular filtration rate (eGFR) [14,15]. We also reported that long-term CR in elderly CVD outpatients helped to maintain the anaerobic threshold (AT), left ventricular EF (LVEF), plasma levels of brain natriuretic peptide (BNP) and eGFR for 5 years [16]. These studies indicate that CR is effective in patients with hypertension (HTN), CKD and/or advanced age. Thus, CR may be effective for HFpEF patients caused by various factors, such as HTN, CKD and/or aging.

It is confirmed and clearly evidenced that CR is associated with the prognosis of HFrEF patients, whereas the evidence in HFpEF patients is relatively weak. Recently, it has been reported that CR improves the exercise capacity and QOL of HFpEF patients [18,19,20], and it has been suggested that it also improves the prognosis of these patients [21]. In the real world, many patients begin, but later discontinue, CR for various reasons. We previously reported that continuation of a 5-month CR program was associated with the prevention of all-cause death and hospital admissions in outpatients with CVD including HF [17]. Therefore, we hypothesized that the continuation of CR would have a positive effect on the prognosis for HFpEF patients and aimed to compare the clinical outcomes in HFpEF patients to those in non-HFpEF patients who did and did not continue a 5-month CR program.

## 2. Materials and Methods

### 2.1. Study Population and Protocol

This single-center retrospective cohort study evaluated the efficacy of CR in HFpEF and non-HFpEF patients. One hundred and seventy-three outpatients who had HF and participated in a 5-month CR program at Fukuoka University Hospital were enrolled from 2011 to 2019. We divided the patients into two groups according to LVEF at the beginning of the CR program: ≥50% LVEF (*n* = 84, HFpEF group) and <50% LVEF (*n* = 89, non-HFpEF group). In addition, we further divided the patients into those who continued the 5-month CR program (continued group) and those who did not (discontinued group). The clinical outcomes were compared between the groups for up to 5 months. The primary endpoint was all-cause death and hospital admission. This study was approved by the ethics committee of Fukuoka University Hospital.

### 2.2. Exercise Protocol

At the beginning of the CR program, the patients underwent a cardiopulmonary exercise test (CPX) using Cpex1 (Inter Riha, Tokyo, Japan) and a stress test system with an ML9000 electrocardiogram (Fukuda Denshi, Tokyo, Japan). The CR program was the same as in our previous report [15]. In brief, the patients participated in a supervised exercise training program at the hospital’s gym one to three times a week. The exercise workload was based on the AT according to the CPX results. Each session lasted 1 h, beginning with a warm-up exercise for 5 min, followed by 30 min of cycling with an aero bike (75XLIII; Combi, Tokyo, Japan) or walking on a treadmill (TRD-350; Sakai Medical, Tokyo, Japan) at the patient’s indicated exercise intensity and 25 min of cooling down and stretching.

### 2.3. Data Collection

Patient characteristics, including comorbidities, medications, LVEF and plasma BNP, were assessed at baseline. Patients with low-density lipoprotein cholesterol ≥140 mg/dL, triglyceride ≥150 mg/dL or high-density lipoprotein cholesterol <40 mg/dL and lipid-lowering therapy were diagnosed with dyslipidemia (DL). Patients with systolic and/or diastolic blood pressure (SBP/DBP) ≥140/90 mmHg or who were under antihypertensive treatment were considered to have HTN. Patients who were being treated for diabetes mellitus (DM) or who had symptoms of DM and a hemoglobin A1c ≥6.5% and/or a fasting glucose concentration ≥126 mg/dL were considered to have DM. Otherwise, the results of a 75 g oral glucose tolerance test were used to diagnose DM. Coronary artery disease (CAD) was defined as lumen diameter stenosis >50% in at least one major coronary artery as determined by coronary angiography and as diagnosed by anterior myocardial infarctions. Medications included angiotensin converting enzyme inhibitor (ACE-I)/angiotensin II receptor blockers (ARB), β-blockers, diuretics, calcium channel blockers (CCBs) and statins. LVEF obtained by the modified Simpson’s method was analyzed by a Vivid E9 echocardiogram (GE Healthcare, Tokyo, Japan). BNP was analyzed via enzymatic methods in the clinical laboratory of Fukuoka University Hospital.

### 2.4. Statistical Analyses

All of the data analyses were performed using the SAS (Statistical Analysis System) Software Package (Ver. 9.4, SAS Institute Inc., Cary, NC, USA) at Fukuoka University (Fukuoka, Japan). Continuous variables with a normal distribution are expressed as mean ± standard deviation, and differences between groups were compared using a Student’s t-test. Differences in categorical variables between groups were compared by chi-square analysis. To identify the factors associated with all-cause death and hospital admission, we performed a Cox regression analysis and obtained hazard ratios. Age, gender, BMI, CAD and medications were also subjected to a multivariate analysis to identify independent factors related to all-cause death and hospital admission. A Kaplan–Meier analysis (log-rank test) was applied to verify the time-dependent occurrence of clinical outcomes in groups stratified according to whether they did, or did not, complete a 5-month CR program. A value of *p* < 0.05 was considered significant.

## 3. Results

### 3.1. Patient Characteristics at Baseline in All Patients and in the HFpEF and Non-HFpEF Groups

The patient characteristics at baseline are shown in Table 1. For all the patients, the mean age was 66.6 ± 13.0 years. The mean age in the HFpEF group (70.0 ± 12.7 years) was higher than that in the non-HFpEF group (63.7 ± 12.1 years, *p* = 0.001). Although there was no difference in comorbidities except for CAD between the groups, the patients in the HFpEF group had less drug interventions, except for statins, than those in the non-HFpEF group (ARB/ACE-I: *p* = 0.002; diuretics: *p* < 0.0001; β-blockers: *p* = 0.0004; CCB: *p* = 0.0003; statins: *p* = 0.6). The BNP at baseline in all the patients was 390 ± 494.9 pg/mL, and that in the HFpEF group was significantly lower than that in the non-HFpEF group (205 ± 230 pg/mL vs. 558 ± 602 pg/mL, *p* < 0.0001). There was no significant difference in the AT peak VO2 between the groups.

### 3.2. Continuation of CR and All-Cause Death and Hospital Admission in All Patients and in the HFpEF and Non-HFpEF Groups

The continuation of 5 months of CR and all-cause death and hospital admissions in all the patients and in the HFpEF and non-HFpEF groups are shown in Table 2. Of the 88 patients (50.9%) who continued the 5-month CR program, 41 (48.8%) were in the HFpEF group and 47 (52.8%) were in the non-HFpEF group. There was no difference in the % continuation of CR between the HFpEF and non-HFpEF groups (*p* = 0.65). In addition, all-cause death and hospital admissions occurred in 16 patients (19.0%) in the HFpEF group and 19 patients (21.3%) in the non-HFpEF group. There was no significant difference in all-cause death and hospital admissions between the groups (*p* = 0.85).

### 3.3. Patient Characteristics at Baseline in the Continued and Discontinued Groups in All HF Patients and in the HFpEF and Non-HFpEF Groups

The patient characteristics at baseline in the continued and discontinued groups in all patients and in the HFpEF and non-HFpEF groups are shown in Table 3. There were no significant differences in patient characteristics at baseline between the continued and discontinued groups in all the patients, or in the HFpEF and non-HFpEF groups except for the %DM in all the patients and the non-HFpEF group. There were also no significant differences in BNP levels at baseline among the groups.

### 3.4. Kaplan–Meier Curves for All-Cause Death and Hospital Admission in the Continued and Discontinued Groups in All Patients and in the HFpEF and Non-HFpEF Groups

The mean follow-up periods were 136 ± 33 days in all the patients. During follow-up, there were 35 composite outcomes, including 3 all-cause mortalities (1.7%) and 32 hospital admissions (18.5%).

**Figure 1:**
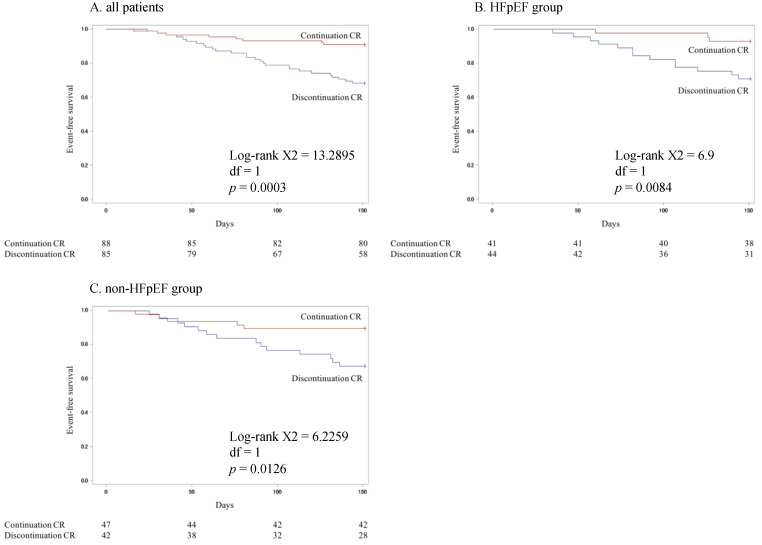
Kaplan–Meier curves for all-cause death and hospital admissions in the continuation and discontinuation groups in all HF patients (**A**) and in the HFpEF (**B**) and non-HFpEF (**C)** groups.

Figure 1A shows the Kaplan–Meier curves for all-cause death and hospital admissions in the continued and discontinued groups in all the patients and in the HFpEF and non-HFpEF groups. The outcome rate was significantly lower in the continued group (*p* = 0.0003). The Kaplan–Meier curves for patients in the HFpEF and non-HFpEF groups are shown in Figure 1B,C. The rates of all-cause death and hospital admissions in the continued HFpEF and non-HFpEF groups were significantly lower than those in the respective discontinued groups (HFpEF group: *p* = 0.0084; non-HFpEF group: *p* = 0.0126).

### 3.5. Relationships between Various Parameters and All-Cause Death and Hospital Admissions as Assessed by a Univariate Logistic Regression Analysis

Regarding the relationships between various parameters, all-cause death and hospital admissions (Table 4), significant relationships were observed for the continuation of the 5-month CR program (all patients: *p* = 0.0007; HFpEF patients: *p* = 0.02; non-HFpEF patients: *p* = 0.02) and the use of diuretics in all the patients (*p* = 0.04).

In Table 5, Model 1 shows unadjusted data. Models 2 and 3 were adjusted for age and age, gender and BMI, respectively. In addition, Models 4 and 5 were adjusted for age, gender, BMI and CAD and age, gender, BMI, CAD and medications, respectively. For Model 5, all-cause death and hospital admissions were independently associated with continuation of the CR program (all patients: *p* = 0.0002; HFpEF patients: *p* = 0.02; non-HFpEF patients: *p* = 0.046) and diuretics in all the patients (*p* = 0.03).

## 4. Discussion

### 4.1. Discussion

The continuation of a 5-month CR program was associated with the prevention of all-cause death and hospital admissions in both the HFpEF and non-HFpEF groups. While it is clearly important to introduce CR, these results suggest that it is also important to continue CR in HF patients, including HFpEF patients.

To date, no pharmacological intervention has been demonstrated to improve the prognosis of patients with HFpEF. Recently, it has been suggested that CR may improve the prognosis of these cases [21]. However, that report simply compared a CR group to a non-CR group. In our study, we were able to extract a group that did not continue CR despite having started CR, which was less effective than in patients who were able to continue CR as planned, at least among HFpEF patients. This supports the notion that the prognosis may be improved by the continuation of CR, especially for HFpEF patients, who are difficult to treat pharmacologically.

CR has been reported to reduce peripheral vascular resistance [22], improve endothelial function [23] and reduce sympathetic tone [24] and to significantly decrease SBP [25]. In fact, our previous report also confirmed that CR lowers BP [14]. CR reduced the visit-to-visit variability in BP, which has been shown to be a strong predictor of CVD and stroke independent of mean BP and other CVD risk factors. Since HTN is one of the main causes of HFpEF, it may have contributed to the prevention of all-cause death and hospital admissions in the HFpEF group. In addition, HFpEF is known as a syndromal disease where multiple cardiac and vascular disorders, cardiovascular risk factors and overlapping extracardiac comorbidities may be present in many kinds of combinations. Specifically, multiple cardiac and vascular disorders mean not only ventricular dysfunction but also vascular dysfunction and valvular disease, cardiovascular risk factors, mean HTN and DM, extracardiac comorbidities, mean renal dysfunction, obesity, sarcopenia and so on. It has been reported that CR has various effects such as inducing an anti-inflammatory response [26], as well as effects on skeletal muscle and the mitochondrial ultrastructure [27,28]. It is possible that the improvement of multiple factors, rather than a single factor, with the continuation of the CR program was responsible for the greater effect in HFpEF patients.

The discontinuation of a 5-month CR program was significantly associated with the presence of DM in all the patients and non-HFpEF patients. However, all-cause death and hospital admissions were not associated with the presence of DM. It was unclear why the CR continuation rate of DM patients was low, but an increase in this rate may lead to an improved prognosis. DM patients frequently discontinue their pharmacological treatment; in a previous study, 12% dropped out after the initial visit to the hospital and 33% of the residual cohort dropped out during each subsequent 6-month period [29]. Multifaceted interventions, which include measuring quality-of-care indicators for patients and providing feedback to physicians, were effective in improving the quality of care and the continuation rate for treatment in patients with DM [30]. Such interventions may contribute to improve the continuation rates for pharmacological treatment and CR in DM patients.

There was no significant difference in the rate of continuation between the HFpEF and non-HFpEF groups (*p* = 0.65). All-cause death and hospital admissions occurred in 16 patients (19.0%) in the HFpEF group and 19 patients (21.3%) in the non-HFpEF group. There were no significant differences in all-cause death and hospital admissions between the groups (*p* = 0.85). A previous study reported that patients with HFpEF and non-HFpEF had nearly identical prognoses [6], which is consistent with our report. This study reaffirmed that HFpEF patients have a poor prognosis over a short time period.

### 4.2. Study Limitations

This study has several limitations. First, this was a retrospective study from a single center with a relatively small sample size. Second, we did not perform long-term follow-ups. Third, the patients received different kinds and doses of medications. Fourth, there were many patients whose reasons for CR discontinuation were unknown. Among 85 patients with CR discontinuation, the reasons for discontinuation of CR were the onset of non-cardiac diseases without hospitalization (*n* = 15), the lowering of motivation (*n* = 15), problems with access to the hospital (*n* = 8), being busy with work (*n* = 4), frailty (*n* = 3), changing to home rehabilitation (*n* = 2), self-will (*n* = 2) and others (*n* = 3). Thus, 33 patients had unknown reasons for discontinuation. It is undeniable that some patients may be too frail or vulnerable to continue CR, and a worse outcome may result from their patient baseline. Therefore, a large-scale long-term study should be performed to confirm these results.

## 5. Conclusions

In this study, the continuation of a 5-month CR program was associated with the prevention of all-cause death and hospital admissions in both HFpEF patients and non-HFpEF patients.

## Figures and Tables

**Table 1:** Patient characteristics at baseline in all HF patients and HFpEF and non-HFpEF groups.

	All HF Patients	HFpEF	Non-HFpEF	HFpEF vs. HFpEF *p* Value
Age, y	66.6 ± 13.0	70.0 ± 12.7	63.7 ± 12.1	0.001
Gender (male), *n* (%)	80 (46.2)	38 (45.2)	42 (47.7)	0.7
BMI, kg/m^2^	23.2 ± 4.3	23.1 ± 4.7	23.3 ± 4.0	0.7
HTN, *n* (%)	88 (50.9)	45 (53.6)	43 (48.9)	0.5
DM, *n* (%)	37 (21.4)	14 (16.7)	23 (26.1)	0.1
DL, *n* (%)	67 (38.7)	32 (38.1)	35 (39.8)	0.8
CAD, *n* (%)	30 (17.4)	9 (10.8)	21 (23.9)	0.03
LVEF, %	46.7 ± 18.7	63.5 ± 7.2	30.8 ± 10.5	<0.0001
Medications				
ARB/ACE-I, *n* (%)	122 (72.2)	50 (61.7)	72 (82.8)	0.002
Diuretics, *n* (%)	124 (73.4)	46 (56.8)	78 (89.7)	<0.0001
β-Blockers, *n* (%)	128 (75.7)	52 (64.2)	76 (87.4)	0.0004
CCB, *n* (%)	44 (26.0)	31 (38.3)	12 (13.8)	0.0003
Statins, *n* (%)	59 (34.9)	30 (37.0)	29 (33.3)	0.6
CPX				
VO_2_ at AT (ml/min/kg)	12.0 ± 3.1	12.7 ± 3.4	11.4 ± 2.6	0.07
Peak VO_2_ (ml/min/kg)	14.9 ± 4.8	15.9 ± 5.1	13.9 ± 4.4	0.07
BNP (pg/mL)	390 ± 495	205 ± 230	558 ± 602	<0.0001

HF, heart failure; HFpEF, heart failure with preserved ejection fraction; BMI, body mass index; HTN, hypertension; DM, diabetes mellitus; DL dyslipidemia; CAD, coronary artery disease; LVEF, left ventricular ejection fraction; ARB/ACE-I, angiotensin II receptor blocker/angiotensin converting enzyme inhibitor; CCB, calcium channel blocker; CPX, cardiopulmonary exercise test; VO_2_ at AT, oxygen uptake at anaerobic threshold; BNP, plasma levels of brain natriuretic peptide.

**Table 2:** Continuation of CR and all-cause death and hospital admissions in all HF patients and HFpEF and non-HFpEF groups.

	All HF Patients	HFpEF	Non-HFpEF	HFpEF vs. HFpEF *p* Value
Continuation of 5-month CR (%)	88 (50.9)	41 (48.8)	47 (52.8)	0.65
All-cause death and hospital admissions (%)	35 (20.2)	16 (19.0)	19 (21.3)	0.85

HF, heart failure; HFpEF, heart failure with preserved ejection fraction; CR, cardiac rehabilitation.

**Table 3:** Patient characteristics at baseline in the continued and discontinued groups in all HF patients and HFpEF and non-HFpEF groups.

	All HF Patients			HFpEF Group			Non-HFpEF Group		
	Cont’d Group	Discont’d Group	Cont’d vs. Discont’d *p* Value	Cont’d Group	Discont’d Group	Cont’d vs. Discont’d *p* Value	Cont’d Group	Discont’d Group	Cont’d vs. Discont’d *p* Value
Age, y	65.5 ± 11.2	67.8 ± 14.6	0.2	68.1 ± 11.3	71.0 ± 14.8	0.3	63.1 ± 10.6	64.4 ± 13.7	0.8
Gender (male), *n* (%)	42 (47.7)	38 (44.7)	0.7	19 (46.3)	19 (43.2)	0.8	23 (48.9)	19 (46.3)	0.7
BMI, kg/m^2^	22.9 ± 4.1	23.6 ± 4.5	0.3	22.8 ± 4.5	23.3 ± 4.8	0.7	22.9 ± 3.7	23.8 ± 4.2	0.2
HTN, *n* (%)	43 (48.9)	45 (52.9)	0.6	22 (53.7)	23 (52.3)	1	21 (44.7)	22 (52.4)	0.5
DM, *n* (%)	12 (13.6)	25 (29.4)	0.01	5 (12.2)	9 (20.5)	0.4	7 (14.9)	16 (38.1)	0.02
DL, *n* (%)	36 (40.9)	31 (36.5)	0.5	17 (41.5)	15 (34.1)	0.5	19 (40.4)	16 (38.1)	0.8
CAD, *n* (%)	17 (19.3)	13 (15.5)	0.5	5 (12.2)	4 (9.3)	0.7	12 (25.5)	9 (22.0)	0.6
LVEF, %	44.9 ± 19.0	48.6 ± 18.4	0.2	62.5 ± 6.9	64.3 ± 7.4	0.3	29.6 ± 10.9	32.1 ± 10.0	0.3
Medications									
ARB/ACE-I, *n* (%)	60 (70.6)	62 (73.8)	0.6	23 (59.0)	27 (62.8)	0.7	37 (80.4)	35 (85.4)	0.7
Diuretics, *n* (%)	61 (71.8)	63 (75.0)	0.6	21 (53.9)	25 (58.1)	0.7	40 (87.0)	38 (92.7)	0.6
β-Blockers, *n* (%)	69 (81.2)	59 (70.2)	0.1	28 (71.8)	24 (55.8)	0.1	41 (89.1)	35 (85.4)	0.4
CCB, *n* (%)	22 (25.9)	22 (26.2)	0.96	15 (38.5)	17 (39.5)	0.9	7 (15.2)	5 (12.2)	0.9
Statins, *n* (%)	33 (38.8)	26 (31.0)	0.3	16 (41.0)	14 (32.6)	0.4	17 (37.0)	12 (29.3)	0.4
CPX									
VO_2_ at AT (ml/min/kg)	12.1 ± 3.1	11.9 ± 3.2	0.8	12.7 ± 3.2	12.8 ± 4.4	0.95	11.4 ± 2.9	11.2 ± 1.9	0.8
Peak VO_2_ (ml/min/kg)	15.0 ± 4.4	14.7 ± 6.1	0.8	15.6 ± 3.8	17.0 ± 8.5	0.7	14.3 ± 4.9	13.0 ± 2.9	0.4
BNP (pg/mL)	433 ± 534	346 ± 450	0.28	183 ± 222	225 ± 239	0.445	630 ± 623	473 ± 574	0.25

HF, heart failure; HFpEF, heart failure with preserved ejection fraction; Cont’d group, continued group; Discont’d group, discontinued group; BMI, body mass index; HTN, hypertension; DM, diabetes mellitus; DL dyslipidemia; CAD, coronary artery disease; LVEF, left ventricular ejection fraction; ARB/ACE-I, angiotensin II receptor blocker/angiotensin converting enzyme inhibitor; CCB, calcium channel blocker; CPX, cardiopulmonary exercise test; VO_2_ at AT, oxygen uptake at anaerobic threshold; BNP, plasma levels of brain natriuretic peptide.

**Table 4:** The relationship between various parameters and all-cause death and hospital admission analyzed by a Cox regression analysis in all HF patients and HFpEF and non-HFpEF groups.

	All HF Patients		HFpEF Group		Non-HFpEF Group	
	HR (95%CI)	*p* Values	HR (95%CI)	*p* Values	HR (95%CI)	*p* Values
Age, y	1.01 (0.98–1.04)	0.4	1.01 (0.97–1.05)	0.7	1.02 (0.98–1.06)	0.3
Gender (male), *n* (%)	1.84 (0.93–3.62)	0.08	1.55 (0.58–4.15)	0.4	2.12 (0.83–5.38)	0.1
BMI, kg/m^2^	0.99 (0.91–1.07)	0.8	1.01 (0.91–1.13)	0.8	0.96 (0.85–1.10)	0.6
HTN, *n* (%)	0.98 (0.51–1.91)	0.96	2.79 (0.90–8.66)	0.08	0.44 (0.17–1.16)	0.1
DM, *n* (%)	1.78 (0.87–3.64)	0.1	1.78 (0.57–5.53)	0.3	1.74 (0.69–4.43)	0.2
DL, *n* (%)	1.03 (0.52–2.03)	0.9	1.69 (0.63–4.50)	0.3	0.67 (0.25–1.76)	0.4
CAD, *n* (%)	0.42 (0.13–1.38)	0.2	1.27 (0.29–5.57)	0.8	0.16 (0.02–1.22)	0.08
LVEF, %	1.00 (0.98–1.01)	0.7	1.00 (0.93–1.07)	0.9	1.00 (0.96–1.05)	0.9
Medications						
ARB/ACE-I, *n* (%)	1.84 (0.76–4.45)	0.2	2.61 (0.76–9.23)	0.1	1.13 (0.33–3.89)	0.8
Diuretics, *n* (%)	3.03 (1.07–8.59)	0.04	2.29 (0.73–7.18)	0.2	N.D.	
β-Blockers, *n* (%)	1.94 (0.75–5.00)	0.2	1.50 (0.69–5.25)	0.5	3.13 (0.42–23.47)	0.3
CCB, *n* (%)	1.00 (0.47–2.14)	1	1.90 (0.69–5.25)	0.2	0.30 (0.04–2.26)	0.2
Statins, *n* (%)	0.54 (0.24–1.18)	0.1	0.80 (0.27–2.35)	0.7	0.36 (0.10–1.22)	0.1
CPX						
VO_2_ at AT (ml/min/kg)	0.98 (0.75–1.28)	0.9	0.98 (0.70–1.39)	0.9	0.98 (0.63–1.54)	0.9
Peak VO_2_ (ml/min/kg)	0.90 (0.73–1.12)	0.4	1.02 (0.84–1.24)	0.8	0.74 (0.50–1.09)	0.1
BNP (pg/mL)	1.00 (0.99–1.00)	0.1	1.00 (0.99–1.00)	0.8	1.00 (0.99–1.00)	0.1
Continuation of 5-month CR	0.26 (0.12–0.56)	0.0007	0.21 (0.06–0.74)	0.02	0.29 (0.11–0.82)	0.02

HF, heart failure; HFpEF, heart failure with preserved ejection fraction; HR, hazard ratio; CI, confidence interval; BMI, body mass index; HTN, hypertension; DM, diabetes mellitus; DL dyslipidemia; CAD, coronary artery disease; LVEF, left ventricular ejection fraction; ARB/ACE-I, angiotensin II receptor blocker/angiotensin converting enzyme inhibitor; CCB, calcium channel blocker; CPX, cardiopulmonary exercise test; VO_2_ at AT, oxygen uptake at anaerobic threshold; CR, cardiac rehabilitation.

**Table 5:** The relationship between all-cause death and hospital admissions and its associated factors for continuation of 5-month CR program and in 5-month period in all HF patients (A) and HFpEF (B) and non-HFpEF (C) groups, analyzed by a multivariate logistic regression analysis.

Factors	Model 1		Model 2		Model 3		Model 4		Model 5	
	HR (95%CI)	*p* Value	HR (95%CI)	*p* Value	HR (95%CI)	*p* Value	HR (95%CI)	*p* Value	HR (95%CI)	*p* Value
(A) All HF patients										
Continuation of 5-month CR program	0.26 (0.12–0.56)	0.0007	0.26 (0.12–0.57)	0.0008	0.25 (0.11–0.56)	0.0007	0.25 (0.11–0.56)	0.0008	0.28 (0.12–0.62)	0.0002
Diuretics	3.03 (1.07–8.59)	0.04	3.04 (1.07–8.64)	0.04	3.23 (1.13–9.19)	0.03	3.79 (1.32–10.87)	0.01	3.57 (1.11–11.50)	0.03
(B) HFpEF										
Continuation of 5-month CR program	0.22 (0.06–0.76)	0.02	0.22 (0.06–0.77)	0.02	0.21 (0.06–0.76)	0.02	0.20 (0.06–0.71)	0.01	0.21 (0.06–0.75)	0.02
(C) Non-HFpEF										
Continuation of 5-month CR program	0.29 (0.11–0.82)	0.02	0.29 (0.11–0.81)	0.02	0.27 (0.10–0.76)	0.01	0.20 (0.06–0.71)	0.01	0.31 (0.10–0.98)	0.046

Model 1. Unadjusted; Model 2. Adjusted for age; Model 3. Adjusted for age, gender and BMI; Model 4. Adjusted for age, gender, BMI and CAD; Model 5. Adjusted for age, gender, BMI, CAD and medications; HF, heart failure; HFpEF, heart failure with preserved ejection fraction; HR, hazard ratio; CI, confidence interval; BMI, body mass index; CAD, coronary artery disease.

## Data Availability

The data that support the findings of this study are available from the corresponding author upon reasonable request.
